# Non headache phenotypes in pediatric age

**DOI:** 10.1186/1129-2377-16-S1-A32

**Published:** 2015-09-28

**Authors:** Massimiliano Valeriani

**Affiliations:** Headache Centre, Ospedale Bambino Gesù, IRCCS, Rome, Italy; Centre for Sensory-Motor Interaction, Aalborg University, Aalborg, Denmark

Although headache represents the main symptom of migraine, it is a very complex disease and can be manifested by a number of other symptoms. This is particularly evident in pediatric age where clinical conditions different from headache can involve children who are already suffering or will suffer from migrainous headache. In the International Classification of Headache Disorders 3^rd^ edition (ICHD-III), these conditions, occurring as repeated attacks with complete remission between episodes, are defined as “Episodic syndromes which may be associated with migraine”. They include “Cyclical vomiting syndrome” (1.6.1.1), “Abdominal migraine” (1.6.1.2), “Benign paroxysmal vertigo” (1.6.2) and “Benign paroxysmal torticollis” (1.6.3). Though not included in the ICHD-III, other clinical entities, such as motion sickness and limb pain, have been associated with migraine. In order to underline the strict relationship between all these non headache symptoms and migraine, they are also known as “migraine equivalents”. We investigated the migraine equivalents prevalence in a large population of children referred to our pediatric headache centre[1]. A total of 1,134 of children/adolescents (73.2% with migraine and 26.8% with tension-type headache) were included. We found that migraine equivalents could equally involve children with either migraine or tension-type headache and that high frequency of headache attacks correlated with migraine equivalents presence. It was concluded that migraine equivalents should not be considered merely as headache precursors, but they are part of the migrainous syndrome. In a more recent study, we showed that anxiety and somatization levels were higher in migraine children with migraine equivalents, as compared to those without migraine equivalents[2]. Our findings, together with those issued from the literature, suggest that in children and adolescents migraine equivalents should be considered as symptoms of the migrainous disease, thus their inclusion among the diagnostic criteria for pediatric migraine/tension-type headache would be hopeful.

## References

[1] Tarantino S, Capuano A, Torriero R, Citti M, Vollono C, Gentile S, Vigevano F, Valeriani M (2014). Migraine equivalents as part of migraine syndrome in childhood. Pediatr Neurol.

[2] Tarantino S, De Ranieri C, Dionisi C, Gagliardi V, Capuano A, Vigevano F, Gentile S, Valeriani M (2015). Migraine equivalents and related symptoms, psychological profile and headache features: which relationship?. J Headache Pain.

